# Active Vision during Action Execution, Observation and Imagery: Evidence for Shared Motor Representations

**DOI:** 10.1371/journal.pone.0067761

**Published:** 2013-06-25

**Authors:** Sheree A. McCormick, Joe Causer, Paul S. Holmes

**Affiliations:** 1 Institute for Performance Research, Manchester Metropolitan University, Crewe, United Kingdom; 2 Brain and Behaviour Laboratory, Liverpool John Moores University, Liverpool, United Kingdom; University of Bologna, Italy

## Abstract

The concept of shared motor representations between action execution and various covert conditions has been demonstrated through a number of psychophysiological modalities over the past two decades. Rarely, however, have researchers considered the congruence of physical, imaginary and observed movement markers in a single paradigm and never in a design where eye movement metrics are the markers. In this study, participants were required to perform a forward reach and point Fitts’ Task on a digitizing tablet whilst wearing an eye movement system. Gaze metrics were used to compare behaviour congruence between action execution, action observation, and guided and unguided movement imagery conditions. The data showed that participants attended the same task-related visual cues between conditions but the strategy was different. Specifically, the number of fixations was significantly different between action execution and all covert conditions. In addition, fixation duration was congruent between action execution and action observation only, and both conditions displayed an indirect Fitts’ Law effect. We therefore extend the understanding of the common motor representation by demonstrating, for the first time, common spatial eye movement metrics across simulation conditions and some specific temporal congruence for action execution and action observation. Our findings suggest that action observation may be an effective technique in supporting motor processes. The use of video as an adjunct to physical techniques may be beneficial in supporting motor planning in both performance and clinical rehabilitation environments.

## Introduction

Movement imagery (MI), the covert rehearsal of human movement, has been proposed to improve motor performance and motor learning in a number of areas, for example, sport [1], [2] and, more recently, rehabilitation [3]. In addition, it has been known for some time that the action observation (AO), also referred to as modelling, can facilitate learning and performance for the observer [4]. Recently, AO has also been shown to support MI for individuals who experience difficulties in generating images [5] and to act as a prime for action execution (AE) [6]. Since there is good evidence for movement optimization via one or more of these three action-related conditions (AE, AO and MI), it is intuitively appealing to propose that both MI and AO may be accessing the same neural substrate as AE and sharing similar mechanisms for motor behaviour. In support for this claim, a growing body of evidence suggests that all conditions, AE, AO [7] and MI [8], are similarly constrained by one of the fundamental laws governing human movement, Fitts’ Law. Specifically, the law states that the time needed to move as quickly as possible between two targets is determined by the width of the targets and the distance separating them [9]. In addition, data from brain imaging studies have revealed interdependence between these action-related cognitive skills linked closely to their neural anatomical-equivalence [9], [10], [11]. This neural ‘sharedness’ was identified as an important marker within Jeannerod’s simulation hypothesis [12]. He proposed the existence of a shared neural network or motor representation that could be accessed to predict action outcome during AE and also generate similar movement planning and expectations during equivalent AO and MI conditions. A common motor representation suggests that the covert elements of action related tasks, intention, programming, and preparation, might be primed and modulated through any of the three simulation conditions, albeit to different extents.

For the past two decades, behavioural changes following AO have been linked to the now ubiquitous human mirror neuron system. This widely distributed network is believed to ‘resonate’ when an individual observes an action that is similar to one held within their own motor repertoire [13]. In line with Jeannerod’s predictions, there is also evidence that motor imagery processes access this frontoparietal mirror network. However, despite the frequency with which the simulation theory is used to explain improvements in motor performance following AO and MI, direct tests of this hypothesis, studies involving all three states in single paradigm, are rare. In addition, the majority of research has primarily focussed on comparisons of neural blood flow. Although the haemodynamic response can be used to indicate cortical activity, a more detailed interpretation of the neuronal activity requires additional evidence acquired through alternative experimental approaches. Therefore, to permit a greater understanding of the simulation theory, it would therefore seem pertinent to include all simulation conditions (AE, AO, MI) in the same experiment and to consider dynamic, behavioural markers.

To date, we have found only one behavioural study [14] where the experimenters compared the three conditions within a single paradigm. In this study, participants were asked to perform, observe and imagine a series of 25 squatting movements whilst lifting a 2.5 kg dumbbell in each hand. Physiological activity was measured during the tasks and compared between conditions. A significant increase in respiration rate, heart rate, and muscle activity, compared to rest, was recorded during their AE condition. In contrast, only respiration rate increased significantly during AO and MI (compared to rest). The authors suggested that mean heart rate might not be sufficiently sensitive to detect subtle change during covert performance. In addition, the results may have been confounded given that perspective was not controlled; AE and MI were conducted from a first person perspective and AO from a third person perspective. In this regard, the observation of the rise and fall of the diaphragm during respiration may have elicited a stronger response in the mirror neuron system during AO.

An emerging, and more sensitive method of comparing overt and covert motor behaviour is eye gaze registration [15], [16]. The gaze metrics commonly measured in this experimental approach are fixations, the brief periods of time (typically greater than 100 ms when the eyes are stable and consciously focused on a visual cue [17]). Fixations can be described in terms of their: (i) duration, suggested to reflect information processing demand; (ii) location, considered to represent visual cue attendance; (iii) number, i.e. how many are made to a target and influenced by skill level and task complexity, and; (iv) movement time, a temporal marker defined as the time between the end of one fixation and the start of the next [18]. Collectively, the fixation metrics permit real-time analysis of cognitive processes associated with visuomotor tasks. Supporting Jeannerodian theory, contemporary research has demonstrated that fixations are congruent between AE and MI [18], AE and AO [19], and AO and MI [20]. Using this fixation approach with a block stacking task, Flanagan and Johansson [19] observed a similar proactive strategy where the fixations pre-empted hand movement in AE and AO. Based on the temporal congruency of the fixations, they reasoned that a motor strategy, rather than a purely visual strategy, is invoked during AO as it is in AE. In contrast, Gesierich et al. [21] employed a similar task and reported that some, but not all, participants executed reactive eye movements where fixations followed hand movement in AO. They proposed that the motor representation was not rigid between conditions but context dependent. The contrasting findings may also be explained by the different task instructions. In the Flanagan and Johansson study [22], no observation instructions were provided. In contrast, Gesierich et al. [21] asked participants to “observe how the model creates the pyramid”. Task instructions that precede AO have been demonstrated to significantly influence subsequent neural activation [23]. Specifically, the instructions to “observe with the intent to imitate” have been shown to activate a neural profile that is most similar to AE. The different gaze strategies may therefore reflect different interpretations of the task requirements. This highlights the need for strict and consistent task instructions and the use of manipulation checks to confirm appropriate task compliance. In a study that controlled task instructions carefully, McCormick et al. [20] considered eye gaze metrics in AO and unguided MI (UGMI; imagery performed in the absence of visual and temporal cues) conditions using a horizontal reach movement. They observed no difference in fixation location, but found a significant difference in fixation duration. They reasoned that the shorter fixation duration in UGMI might have been due to the decrease in information processing demands resulting from reduced visual percepts. In a similar manner, other researchers [18] report that the congruency of fixations between AE and MI can be enhanced when the MI task is assisted with visual and temporal cues. Corroborating these reports, Gueugneau et al. [24] demonstrated that MI was facilitated when visual cues were available and contingent eye movements were permitted (versus prevented). Collectively, these studies imply that similar information is attended to across simulation conditions but not the detail of how eye movement metrics may vary across conditions. A comprehensive inter-condition comparison of fixation duration would address this shortfall.

In other domains, such as sports, law enforcement, medicine and the military, the duration of the final fixation prior to movement onset in aiming tasks is referred to as the ‘quiet eye’ [17]. This period of time is considered to reflect the information processing demands associated with programming the parameters of movement such as force, direction, and velocity. Williams et al. [25] demonstrated that in near aiming tasks (billiards) the quiet eye increases linearly with the complexity of the task. If the quiet eye reflects covert elements associated with motor performance, then, in accordance with simulation theory, it should be comparable (relatively) between simulation conditions and similarly influenced by task complexity.

In the current study, we sought to test directly and comprehensively the simulation theory using eye movements. We compared fixation metrics across four different conditions (AE, AO, guided motor imagery (GMI) and UGMI). All participants performed a reach and point task at three levels of complexity, defined by target width. Movement time (MT) was measured in AE, GMI and UGMI conditions to confirm task compliance. Based on the tenets of the simulation theory we hypothesized that:

- MT would be congruent between AE, GMI and UGMI;
- the number of fixations to the target location would remain congruent in all conditions; and
- total target fixation duration would be congruent across all conditions.

## Methods

### Ethics Statement

The study was approved by the Exercise and Sport Science Ethics Sub-Committee, Department of Exercise and Sport Science, Manchester Metropolitan University.

### Participants

Following purposeful sampling, a homogeneous group of thirteen healthy participants, with normal or corrected to normal vision and no upper limb motor impairment, volunteered to participate in the study. Participants were naive to the hypotheses being tested and supplied written informed consent prior to participation. Handedness was assessed by the Edinburgh Handedness Inventory [26] with all participants scored as right-handed (96.12±4.36). The age range of participants was 51.49±6.01 years. Age related slowing in motor and cognitive performance is suggested to increase dramatically from 60 years [27], [28]. In this study, we compared the performance of adults below this threshold, assuming motor and cognitive skills to be unimpaired.

### Task

**Figure 1:**
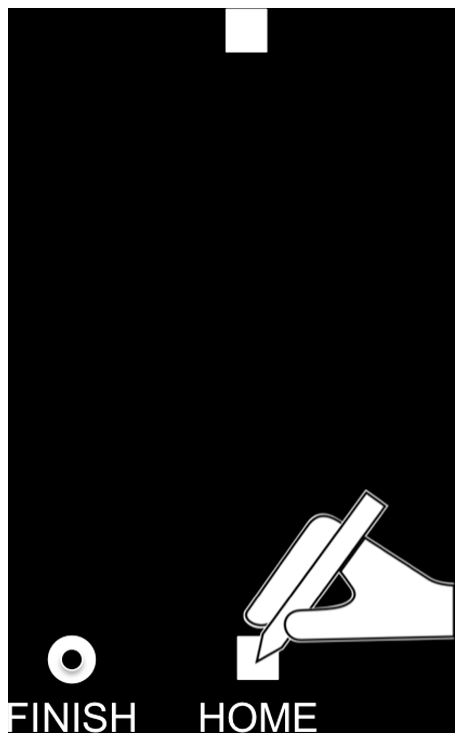
Forward reach and point task. In the action execution task the participants moved the stylus accurately and rapidly from the HOME button to the target, back to HOME and then to FINISH.

Participants performed a modified version of the Virtual Radial Fitts’ Task (VRT) previously employed by others [29], [30]. The task was completed in four conditions: (i) AE; (ii) AO; (iii) GMI; and (iv) UGMI. Participants sat at a table, screened on three sides to occlude task-irrelevant environmental stimuli, and performed a drawing task using a larger touchscreen tablet (ST2220T, Dell UK Inc.) and a hand held stylus (normal pen size and weight) with a thin white tip. On touching the tablet the pen’s movement left a momentary digital trace. During all tasks a HOME and FINISH button was presented on the touchscreen and in AE, AO and GMI (but not UGMI) a TARGET was also present (see Figure 1). The HOME button was presented at a distance of 200 mm away from the participant’s torso (midline). The TARGET was aligned with the HOME button and the amplitude between the closest edges of the HOME and TARGET was constant (185 mm). To vary the complexity of the task three different sized square targets were used; small (4 mm^2^), medium (9 mm^2^) and large (20 mm^2^).

In the AE, GMI and UGMI conditions the participants were required to tap the HOME button as soon as it was presented. Participants then either moved the stylus physically to the TARGET and back to HOME (AE, see Figure 1) or imagined the action without any concomitant movement (GMI and UGMI). In UGMI participants had to imagine both the TARGET position and size. Upon completion of the movement (either overtly or covertly) participants then tapped the FINISH button with the pen. The MT, the time from when the stylus left the HOME button until it tapped the FINISH button, was measured in AE, GMI and UGMI. In AO, the arm was placed outside of a slightly adjusted privacy screen to control for duplication of visual stimuli. In this condition participants observed a recording of their own AE, presented back as a video clip (see apparatus). In all conditions the torso, arm, hand and stylus remained in a similar position.

To ensure a maximally homogeneous task across all participants and conditions the following specific instructions were explained and repeated at the start of each block of trials. In the AE condition, participants were asked to move the stylus as accurately and as quickly as possible. In the GMI and UGMI conditions, participants were instructed to image the task from a first person egocentric visual orientation and to employ both visual and kinaesthetic imagery modalities. The task was to be executed in the same manner as previously performed. Participants were provided with generic stimulus and response propositional instructions [31] associated with the task: “see yourself accurately reach the square target, as if you were actually performing the movement” and “feel your grip on the stylus, feel the muscles in your upper arm contract, feel your arm extend as you perform the movement”. In the observation condition, the participants were instructed to “observe the action with the intention to imitate it at a later time”. In all covert conditions participants were requested to make no physical upper limb movement.

A control condition was included to ensure that the gaze metrics in the simulation conditions were task related. Participants sat at the desk holding the stylus and were instructed to count back slowly from 100; no other task instructions were given. During this time the TARGET, HOME and FINISH buttons were presented on the touchscreen and eye movements were collected. After 50 s (a time equivalent to that spent performing the tasks in AE) the participants were informed the task was complete and were asked to rest.

### Apparatus

The touchscreen and stylus were calibrated pre-experiment. The stylus movements were recorded at 50 Hz using DMDX [32]. The touchscreen had a spatial accuracy _±_2.5 mm, over 95% of touchable area and a typical response time of 15 ms.

Eye movements were recorded with the Applied Science Laboratories Mobile Eye system (ASL; Bedford, Massachusetts) at a sampling rate of 30 Hz. The system has an accuracy of 0.5° of visual angle, a resolution of 0.10° of visual angle, and a visual range of 50° horizontal and 40° vertical. A laptop (Lenovo T500 ThinkPad) installed with ‘Eyevision’ (ASL) recording software was incorporated with the system. The experimenter and laptop were positioned to the right of the privacy screen to minimize visual distraction during all conditions.

The Mobile Eye was calibrated prior to each block of tasks using a 9-point grid presented on the touchscreen. A chin rest was used to restrict head movements and participants were requested to limit both head movements and speech during the experiment. These controls enabled optimal collection of gaze metrics. The eye movement data was analysed using Gazetracker software [33]. As the task consisted of a simple arm extension/flexion movement in the sagittal plane, only vertical gaze was analysed. Pilot testing of the task revealed no significant horizontal eye movements. A fixation was defined as a stable gaze position (i.e., within 0.67° visual angle) that was maintained for at least 120 ms. ‘Look-zones’, areas equivalent to the target size plus a tolerance: small = 8 mm^2^; medium = 7 mm^2^; large = 6 mm^2^, were determined during pilot testing and reflected the area most heavily populated by fixations during the current control phase of the physical movement. The tolerance accommodated for drift, compressions, expansions and individual gaze behaviour preference [34]. Individual look-zones were overlaid onto each target during post processing and all fixations within these zones were considered to be task related. A similar method of spatially comparing fixations between simulation states has been employed by others [34], [35].

To maintain strict intra-individual congruency across all conditions, each participant’s AE trials were filmed using a Sony High Definition Handycam (HDR-HC7E). The camera was positioned directly above the participant and 186 cm from the floor. The filming process was not explained to the participants until after the final debrief session. It was covertly operated during AE with a remote device. The personalized videos were then replayed in the participant’s AO trials.

### Experimental Procedure

The imagery ability of each participant was assessed using the Movement Imagery Questionnaire Revised (MIQ-RS) [36]. The MIQ-RS comprises 14 imagery items; seven visual (VIS) and seven kinaesthetic (KIN). Ease of imagining ratings for each item on the MIQ-RS are selected from a 7-point Likert-type scale where 1 = very hard to see/feel and 7 = very easy to see/feel. Possible scores therefore range from 14 ( = extremely poor imager) to 98 ( = extremely good imager) for the combined modalities.

Following imagery assessment, participants performed a single habituation block of the VRT using a target that was a different size (15 mm^2^) to the experimental tasks. Participants were then assigned to one of three series defined by target size (small, medium, large). Each series began with one block of AE, followed by one block of each of the other conditions (i.e., AO, GMI, UGMI and Control, counterbalanced). Preceding the covert conditions with AE was a necessity to maintain equivalent self-referent representations based on stored memories of a prescribed task [37]. Each block consisted of eleven repetitions of the task followed by a 2 minute rest. The first trial in each block was discarded since pilot testing revealed MT in this trial to be more variable. Excluding the control condition, which was analysed at a block level, 120 trials were analysed for each participant: 10 (tasks per block)×4 (conditions)×3 (target size). At the end of the trials each participant was debriefed fully and completed a self-rated evaluation of their visual and kinaesthetic performance during AO and MI. An in-house questionnaire, using a 7-point Likert-type scale (similar to the MIQ-RS), was used to rate the ease/difficulty associated with their visual and kinaesthetic performance (in the GMI and UGMI conditions) and their active visual engagement and kinesthesis (in AO).

### Dependent Variables

#### Chronometry measures

Performance on the VRT was measured by comparing each participant’s mean MT. The AO condition was excluded from this analysis as no MT data was recorded. If effective MI was performed MT would be comparable between MI and AE and both GMI and UGMI conditions would be influenced by task complexity.

#### Number of fixations

The total number of fixations inside the look-zones (per block of ten trials) was calculated and compared between conditions. Based on the work of Flanagan and Johansson [19], no significant difference in the number of fixations to the target zone would indicate the execution of a similar visual, but not necessarily motor, strategy between conditions. In addition, repeated fixations at the target would provide a measure of participants’ engagement in the covert tasks [38]. The control condition was also included in this analysis. The fixations in this condition were expected to be epiphenomenal rather than functional, and significantly different to that of the simulation conditions [38].

#### Fixation duration

The total fixation duration at the target (per block of ten trials) was computed for each participant and compared between conditions. Comparable values for fixation duration, that were similarly influenced by task complexity, would provide evidence of a shared eye motor program directed by the motor representation for a reach and point action [19].

#### Imagery ability and manipulation checks

The scores of the MIQ-RS were calculated to ensure that all participants presented with at least average MI ability. The manipulation checks were used to confirm appropriate participant compliance in all covert tasks.

### Statistical Analyses

All values deviating more than two standard deviations from the mean were removed. The Shapiro-Wilks and Levene’s test were used to identify normal distribution and equivalent variance. Sphericity was assumed if Mauchly’s test of sphericity was >0.05. Effect sizes were calculated using partial eta squared values (η_p_^2^) and the alpha level for significance was set at 0.05. Pairwise comparisons were LSD corrected. All data are presented as means and Greenhouse-Geisser corrected.

## Results

### Chronometry Measures

To confirm participant task compliance, MT was recorded and compared using a 3 (condition: AE, GMI, UGMI)×3 (target size: small, medium, large) repeated measures (RM) ANOVA. Main effects were found for condition (F_1.403, 16.831_ = 9.338, *p* = 0.004, η_p_^2^ = 0.438, and target size (F_2, 24_ = 3.793, *p* = 0.037, η_p_^2^ = 0.240). There were no significant interactions. Pairwise comparisons revealed MT was significantly quicker in AE (2.681 s) when compared to GMI (3.106 s, *p* = 0.046) and UGMI (3.460 s, *p* = 0.004). MT was also significantly quicker in GMI compared to UGMI (*p* = 0.008). For target size, MT was significantly quicker for the large target (2.943 s) compared to the small target (3.207 s, *p* = 0.028) across all conditions. There was no significant difference between the large and medium targets (3.097 s, *p* = 0.140) or between the medium and small targets (*p* = 0.215).

### Total Number of Fixations

**Figure 2:**
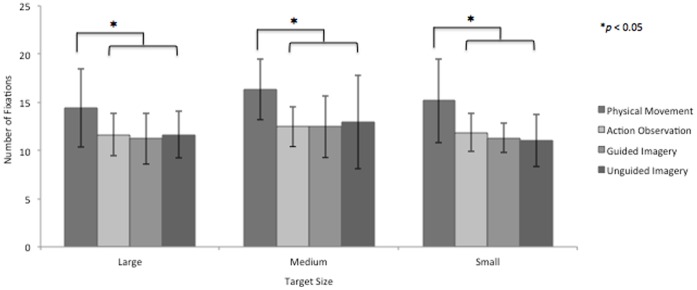
Fixations at the target look-zone. Total number of fixations at the target look-zone during all 10 trials, for all series and conditions.

A 5 (condition: AE, AO, GMI, UGMI, Control)×3 (target size: small, medium, large) RM ANOVA was used to compare the total number of fixations at the target zone. A main effect was found for condition (F_4, 48_ = 21.401, *p*<0.001 η_p_^2^ = 0.641, but not for size (F_2,24_ = 1.527, *p* = 0.113) and there were no interactions. Pairwise comparisons revealed that significantly more fixations were made during AE (15) compared to all other simulation conditions; AO (12, *p* = 0.006), GMI (12, *p* = 0.003), and UGMI (12, *p* = 0.018). There were no significant differences between the covert conditions (see Figure 2).

A control condition was included in this analysis to confirm that the fixations in the target zone were task related. Significantly fewer fixations (5, *p*<0.001) were observed during Control compared to AE, AO, and GMI and UGMI. In addition, the number of fixations in this condition was highly variable as reflected in the large standard deviations (large target 7±6, medium target 5±4, small target 4±5).

### Total Fixation Duration

**Figure 3:**
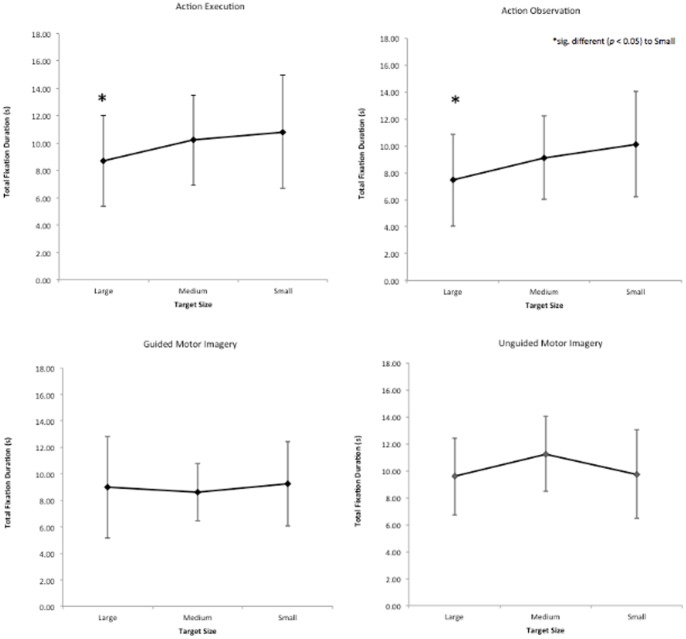
Fixation duration. Total dwell time at target during all 10 trials, for all series and conditions.

A 4 (condition: AE, AO, GMI, UGMI)×3 (size: small, medium, large) RM ANOVA was used to compare total fixation duration. A main effect of size (F_2, 24_ = 4.204, *p* = 0.027, η_p_^2^ = 0.259), but not condition (F_1.603, 19.239_ = 1.656, *p* = 0.194, was observed. There was also a significant size by condition interaction (F_6, 72_ = 2.227, *p* = 0.050, η_p_^2^ = 0.157), see Figure 3. Simple effect analyses revealed that in AE and AO total fixation duration was significantly shorter for the large target size compared to the small target size (AE, 8.692 s vs 10.815 s, *p* = 0.005; AO, 7.447 vs 10.212 s, *p* = 0.001) and for the large target size compared to the medium target size (AE, 8.692 s vs 10.210 s, *p* = 0.002; AO, 7.447 s vs 9.115 s, *p* = 0.054). All other comparisons were not significant.

### Imagery Ability

Participants completed the MIQ-RS to assess ease of imagery generation ability. All participants rated their ability as at least average. Mean scores of 33.82±9.42 (VIS) and 34.09±8.56 (KIN) were recorded. Manipulation checks were performed post experiment to examine participants’ MI and AO experiences. For MI, mean scores (based on the reach and point task only) revealed that the visual component of the imagery was considered at least “*somewhat easy to see*” (GMI = 5.54±1.13, UGMI = 5.47±1.98). Kinesthetic imagery was rated as “*somewhat hard to feel*” (GMI = 3.54±1.11, UGMI = 3.08±1.98). For AO, mean scores revealed that the visual component of the AO was considered at least “*very easy to actively engage with*” (6.77±0.60). The kinesthesis associate with AO was rated as “*very hard to feel*” (1.46±2.82).

## Discussion

The current study explored eye gaze behaviour across four experimental conditions using a forward reach and point Fitts’ Task. Our hypotheses were partially supported; (1) MT was not strictly congruent between AE, GMI and UGMI conditions, but was similarly influenced by target size across all conditions; (2) the number of target fixations was significantly different between AE and all covert conditions but all conditions were similarly influenced by target size; (3) total target fixation duration remained congruent in AE and AO and both conditions displayed an indirect Fitts’ Law effect. The findings suggest there are similarities in the fixation metrics and also some specific differences. Therefore, these data provide partial support for the common representation hypothesis and, for the first time, through eye movement metrics. The discussion is organised by dependent variables.

### Equivalence in Chronometry Measures

The time required to physically perform and imagine a reach and point task was compared in three levels of task difficulty. In agreement with others [8], [39], MT was significantly quicker in AE compared to GMI and UGMI conditions. Decety et al. [8] reasoned that increased force (effort) in AE (required to maintain similar levels of performance in more effortful tasks), is interpreted as increased MT in imagery (of the same task). In the current study, the participants were asked to perform all tasks optimally, i.e. to focus on both speed and accuracy. We suggest that increased effort was required to decelerate the limb and place it accurately and quickly at the medium and small targets during the current control phase of the movement [40], [41]. This increase in effort could have been interpreted as an increase in time during imagery. We also observed that MT was significantly longer in UGMI than GMI. Kosslyn [42] suggests that additional time is required in visual imagery when the tasks are more complex. Our data support this idea given that the UGMI condition required participants to generate and inspect additional images (i.e. the target).

Some researchers have argued that overestimated time in imagined movement can occur because tacit knowledge is used instead of imagery [43], [44]. For example, the participant may count internally to direct the behaviour in an attempt to match the MT. If the participants had used tacit knowledge in the current study, MT should have been similar in both GMI and UGMI conditions. This was not observed since MT in GMI was significantly quicker compared to UGMI.

Fitts’ Law has been demonstrated to constrain MI in a similar manner to AE [8], [39]. In support of the findings of Maruff et al. [39], we found that MT for both physical and imagined conditions was significantly greater for the small, more complex target task compared to the medium and large, less complex target tasks. This data provides further support for the theory of a common motor representation that is accessed during MI and physical execution. In addition, the data confirm participants’ engagement in both MI tasks.

### Number of Fixations

The control condition data demonstrated that the fixations performed during the task were functional rather than epiphenomenal. Participants demonstrated a variable eye movement pattern in this condition (evidenced through the large standard deviations) that was inconsistent with the other experimental conditions. This provided confidence that the eye movements were task related in AE, AO, GMI and UGMI conditions.

There were significantly more fixations in AE compared to the covert conditions. The goal of the task was to point to a target as accurately and quickly as possible, in AE this included the coupling of two effectors, the limb and the eyes. During the AE of a pointing task an individual will typically produce anticipatory saccades to the target site before the limb arrives and remain there until the task is complete [31]. Online comparison of the feedforward efferent motor command with visual and proprioceptive afferent feedback occurs to place the limb accurately at the target. If the placement of the first fixation provides incorrect or insufficient information, a corrective saccade and subsequent fixation occurs [40]. In contrast to AE, in the covert conditions no additional fixations related to error correction of the limb trajectory were required and hence the number of fixations was less in this conditions. The results contradict those of Heremans et al. [18], who reported no significant difference in the number of fixations between AE and visually assisted MI, this may be due to differences in task complexity. In the Heremans et al. study, the relatively simple task involved a controlled cyclic wrist extension/flexion movement, whereas in the current study participants optimally performed (i.e. considered speed and accuracy) a gross motor movement. Our findings highlight the fact that the neural overlap between conditions is not complete. For this metric, the covert conditions have no need, or appear unable, to simulate the fine adjustment of the motor program that occurs in tasks that are guided by afferent feedback and sensory expectation.

The number of fixations in the target zone in UGMI condition was consistent with the other more visually assisted conditions. During object related perception, spatial information is encoded in a spatial index associated with eye movements. It is suggested that later access to the stored representation, either during memory retrieval or imagery, can be achieved by re-executing the same eye movements [38], [45], [46]. In the current study, the UGMI condition provided no task relevant information except the stationary limb and the HOME button. Jeannerod proposed that in visuomotor tasks the motor program in permanently fed with information from two sources, conceptualised as a visual map and a proprioceptive map [47]. The visual map encodes the position of the target with respect to the body using retinal information; the proprioceptive map encodes the static and dynamic proprioceptive signals from the limb. It is therefore possible that the stationary limb provided the motor program with the necessary inputs to be able to re-execute the accurate landing position of the target fixation.

In agreement with others [38], [40], the number of fixations to the target was not influenced by task complexity (i.e. target width). The physical execution of simple aiming tasks typically requires no more than two fixations at the target in order to determine the target’s location in the visuomotor work space [40]. A comparable fixation pattern was observed in this study and implies that the target location was optimally determined in all conditions. This suggests that in overt and covert conditions the number of fixations remains relatively robust to changes in task complexity and provides further evidence of a shared motor program.

### Total Fixation Duration

Fixation duration is reported to consist of three processes: visual field sampling, analysis of foveal information, and planning of the next saccade [48]. In the current study, visual field sampling was controlled using a privacy screen, and planning of the next saccade, after target acquisition, was controlled with the HOME button that was constant across trials. Consequently, it was inferred that any difference in fixation duration was due to the analysis of foveal information. In AE and AO, the fixation duration was significantly influenced by target size; fixation duration was longer for the small compared to the large target. These data indicate that visual processing increased as a function of task complexity for these conditions only.

Some authors [49]–[51] suggest the scaling of the MT by task complexity is an emergent process; crude motor plans are formed during action preparation and continually updated during action performance. The online modulation of the motor output is achieved via the posterior parietal cortex that acts as a ‘neural comparator’, comparing eye signals (retinal and extra-retinal) to proprioceptive and efferent copy signals. In the current study, fixation duration was influenced by task complexity in AE and AO, but not in GMI or UGMI. This suggests that the amount of visual processing is also an emergent process. We propose that the internal, top down model used in imagery is sufficient to generate crude motor plans (the MT was significantly different to AE but similarly influenced by task complexity) but is unable to simulate the dynamic feedback conditions. In contrast, the similar and dynamic behaviour of fixation duration in AE and AO suggests the activation of an enhanced, dynamic motor program that is shared by both conditions.

Other temporal fixation markers (e.g. the time between successive fixations), are also reported to demonstrate no effect of task complexity in cyclic aiming tasks [18]. Combined with our results, these findings suggest that the sub-components of aiming tasks are processed differently in GMI and UGMI compared to AE and AO. These data corroborate the findings of Calmels et al. [52] who reported that elite gymnasts, with medium – high imagery ability, imagined a complex gymnastic routine in a temporally different format to that displayed in physical performance.

The lack of a main effect in fixation duration between conditions (with the target complexity data collapsed) appears to contrast with the findings of others [20]. McCormick et al. [20] reported fixation duration to be significantly longer in AO compared to MI conditions. These differences can be reconciled if the task designs are considered. In the McCormick et al. study, MT was fixed between the AO and MI conditions. In the present study, MT was self-determined during the MI conditions and shown to be significantly longer than AE (and by extension AO, since AO included the video recording of AE). Therefore, in agreement with McCormick et al., this suggests the time spent fixating in MI, relative to MT, was less than the time spent fixating in AE and AO, relative to MT.

### Imagery Ability and Manipulation Checks

All participants were initially assessed as having at least average kinaesthetic and visual imagery skills. The manipulation checks revealed that participants found the visual kinaesthetic component of the imagery more difficult to perform than the visual component. The ability to maintain the temporal preservation of the organization of movement during MI has been taken as evidence of kinaesthetic imagery [53]. It is possible that the overestimation of MT is indicative of the sub-optimal kinaesthetic imagery performances. In contrast, the preservation of spatial information in imagery, evidenced through repeated fixations in the target zone, corroborates the above average scores recorded for visual imagery.

The manipulation checks for AO suggest participants found it easier to engage in this task, compared to the MI tasks. In this condition no instructions to ‘feel’ the movement were issued and the low scores for kinesthesis suggest participant compliance. Thus, it appears that the temporal preservation of the movement in AO, as evidenced through fixation duration, is achieved via mechanisms other than kinesthesis. This could be an eye motor program that is shared in AE and AO.

### Conclusion

In this study, we used a single experimental paradigm and measured eye movements to test the predictions of Jeannerod’s simulation hypothesis [54]. All participants fixated the target look-zone, indicating that similar information was attended to across conditions. Fixation duration was influenced by task complexity in AE and AO only. This suggests that dynamic manipulation of the motor representation in response to task constraints occurs similarly in AE and AO but not MI. As such, AO may be a more effective technique in supporting complex motor processes. The close similarity between AE and AO may support the use of AO as a prime to MI, if chronic immobility has compromised effective physical movement (e.g., stroke). This research highlights the importance of considering the dynamic nature of the motor representation and its influence on behaviour in the covert conditions.
